# An investigation on the predominant diseases, its diagnosis, and commonly used drugs in the poultry farms in the North-Eastern regions of Algeria

**DOI:** 10.14202/vetworld.2018.986-989

**Published:** 2018-07-24

**Authors:** Amine Berghiche, Tarek Khenenou, Ahmed Kouzi, Ibtissem Labiad

**Affiliations:** 1 Department of Veterinary Science, Institute of Agronomic and Veterinarian Sciences, University of Mohamed Cherif Messaâdia, Souk Ahras, Algeria; 2 Laboratory of Animal Production, Biotechnology and Health, University of Mohamed Cherif Messaâdia, Souk Ahras, Algeria; 3 Laboratory of Science and Technique of Living, Institute of Agronomic and Veterinarian Sciences, University of Mohamed Cherif Messaâdia, Souk Ahras, Algeria

**Keywords:** Algeria, diagnosis, disease, investigation, poultry

## Abstract

**Aim:**

An investigation was carried out to assess the occurrence of diseases, its method of diagnosis, and commonly used drugs in poultry farms in North-Eastern regions of Algeria.

**Materials and Methods:**

A total of 265 veterinary doctors were surveyed to obtain information on the dominant diseases, its frequency of occurrence, method of diagnosis, and commonly used drugs in poultry farms.

**Results:**

A study revealed that about 68% of bacterial diseases are due to colibacillosis, mycoplasmosis, and salmonellosis, 22% of viral diseases are due to Newcastle, Gumboro, and infectious bronchitis, and 10% others including coccidiosis and ascites syndrome. The study also showed that about 57% of cases were diagnosed by clinical signs, 36% by necropsy findings, and the remaining 7% through therapeutic and laboratory analysis. Antibiotics, a predominance of the anarchic veterinary drugs, were massively used to control the diseases. Hence, there is a need for strict regulations on the use of veterinary drugs to guarantee food safety.

**Conclusion:**

These results remain non-exhaustive but contribute strongly to determine the status of health of the birds in the region.

## Introduction

Data on the incidence of diseases in poultry reveal important information about the status of that industry, especially in developing country like Algeria. Although the relative importance of diseases may differ between the countries and its geographical areas, there are few important diseases which are unique to particular parts of the world. Figures relevant to the U.S.A. indicated that the total economic loss from disease is about 20% of the value of poultry production, which is about 3 times the loss of mortality. It is considered that infectious disease will continue to be the major cause of economic loss from the disease. Diagnosis of infectious diseases in poultry can be made on the basis of history, clinical symptoms, and presenting pathology. The future developments in disease control measures will use the genetic engineering techniques from the production of vaccines, developing stocks resistant to disease [1], and the curative treatment with antibiotics. There are many important bacterial, viral, parasitic, and metabolic diseases which can affect poultry.

Many preparations exist and can treat bacterial infections. In addition to their curative role, antibiotics were formerly used for prophylaxis or as growth promoter; their use is nowadays restricted to the treatment of bacterial infections. The cost of this practice not only adds cost but also involves a precise diagnosis to avoid any therapeutic failure, economic loss related to the cost of treatment, the decrease in zootechnical performances, and the lengthening of the breeding period to respect the waiting periods. Khnenou *et al*. [2] structured approaches for diagnosis of bacterial infections which are being referred widely by field veterinarians. It is the key to success of any treatment both human and animal disease conditions and any mistake can lead to fatal consequences. Early diagnosis can limit or even avoid substantial economic losses to the farmers by reducing the cost of treatment, morbidity, and mortality [3]. However, accurate diagnosis requires confirmation through laboratory investigations, because of a variety of clinical presentations and especially common occurrence of latently or unapparent subclinical infection, no single diagnostic test can reliably determine infection in poultry farms [4].

A precise procedure including clinical or necropsy examination of birds, the determination of the treatment of choice based on sensibility (antibiogram) and could help the field veterinarians to most adapted proper treatment. In this study, a survey was conducted to evaluate the occurrence of diseases, diagnostic methods used, and selection treatment criteria by the field veterinarians, and poultry farmers. The information generated from this study could serve as principal methods for disease incidence, epidemiology, susceptibility of breeds, control measures, precise treatment, and establishment of preventive measures such as vaccination schedule and identification of disease resistant breeds.

## Materials and Methods

### Ethical approval

The experiment was carried out according to the National Regulations on Animal Welfare and Institutional Animal Ethical Committee.

### Informed consent

The questionnaire was designed to ensure all the elements of regulatory bodies and the individual respondent identity was secretly maintained.

### Experimental design

The study was carried out in the North-Eastern Algeria over a period from January 2015 to June 2017. The survey was based on a questionnaire sent to veterinary practitioners in the poultry sector in the region. Questions have been made to collect information, divided into three main components, which were causal approaches that contained a set of questions that allow us to study the main pathologies encountered in poultry farms, according to certain epidemiological parameters, type, and importance of breeding, etc. Major causatives, their occurrence frequency, and diagnostic techniques employed. The questionnaire also contained priorities of the practitioners with respect to their involvement in poultry activities. The therapeutic approaches followed to understand the self-medication phenomena in the region.

### Statistical analysis

The collected data are logged and processed using the program (Microsoft Office Excel 2007) to perform the description and evaluation.

## Results and Discussion

On the basis of the questionnaires, which have been returned to us, all the information received were taken into consideration to obtain a better idea about how field veterinarians approach their diagnosis and treatment.

### The importance of poultry activity

**Figure-1:**
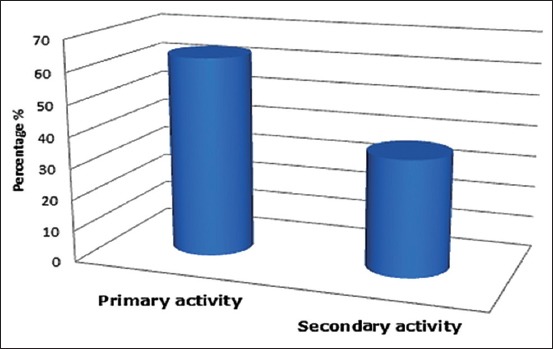
Veterinary practitioner’s involvement in poultry activities in the region.

About 63.26% of veterinarians in the region were involved in poultry as their primary activity, while the remaining 36.74% involves them as a secondary activity (Figure-1). The results revealed that about two-thirds of veterinary practitioners were involved in the poultry activities.

The results of our investigation are very close to that obtained by Messai [5], who found that the poultry clientele activity is dominant in 63% of veterinarians.

### Diagnosis methods

**Figure-2:**
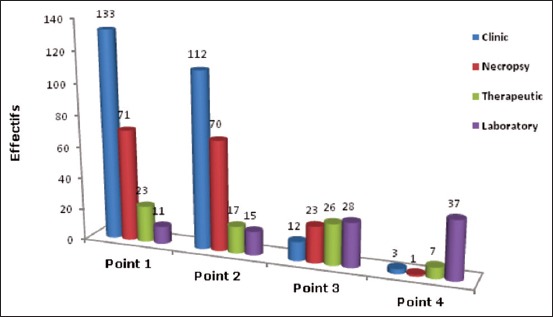
Diagnostic methods and their frequency.

The diagnostic methods could be related to stakeholder experience. We observed that about 57% of conditions were diagnosed by their clinical signs along with their frequency of occurrence. The diagnosis is also followed by necropsy findings (36%), response to therapeutic approaches (05%), and laboratory diagnosis (02%), (Figure-2).

Among various diagnostic techniques, the laboratory diagnosis method is least used (02%). This could be due to the inaccessibility of the regional laboratories by field veterinary interlocutors. Moreover, the laboratories were too late to deliver the results and due to the lack of sufficient chemicals and other related analytical tools [6].

A huge number of veterinarians have to apply the clinical diagnostic methods in poultry farms. However, in the eastern area of Algerian, the majority of practitioners (86%) often use clinical diagnosis as the only diagnostic method based on clinical symptoms and lesions (necropsy examination). Most of the poultry diseases exhibit similar clinical signs and necropsy findings which may often mislead in diagnosis of diseases. Hence, the use of polymerase chain reaction-based detection should be followed as a standard technique for the detection of avian diseases. It is also believed that only part of our practitioners uses the response to treatment as a toll of diagnosis [6,7].

### Most important diseases reported in the region

**Figure-3:**
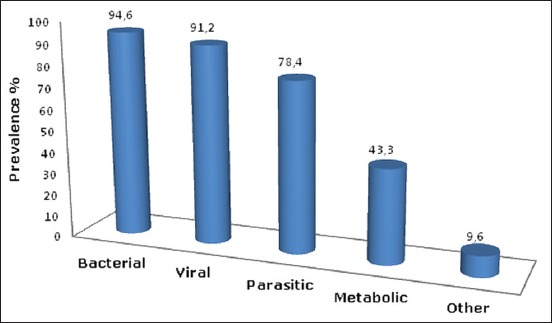
The frequency of diseases in poultry farms.

Figure-3 represents the percentage of the type of diseases occurred. We encountered 94.6% of bacterial diseases, 91.2% of viral diseases, 78.4%, 42.3%, and 9.6% for parasitic, metabolic, and other diseases, respectively.

### Most common bacterial diseases

The most important bacterial diseases in poultry farms were colibacillosis (49%), followed by mycoplasmosis (45%), salmonellosis (4%), and coryza infectious (2%). Since 2009, colibacillosis becomes the most reported disease in broiler chickens in RNOEA [8]. In other countries, particularly in western Europe, over the past few years, a resurgence of colibacillosis has been observed in the laying hens’ sector [9,10]. In addition, according to a study carried out in English slaughterhouses, 43% of carcasses seized due to pericardium, périhépatite, and aérosacculite typical lesions of colibacillosis [11].

The results of these studies are consistent with our results. This is further explained that the digestive tract is the probable reasons, as it acts as the important reservoir of avian *Escherichia coli*. It has been reported that the dust particles present in the farms contain up to 10^6^ coliforms per gram and the serotypes found the similar results in septicemia lesions [12].

The study also highlighted the importance of the mycoplasmosis in birds and also reported by two separate studies carried out in Batana area in 2006 and 2011 [13,14].

### Most common viral diseases

**Figure-4:**
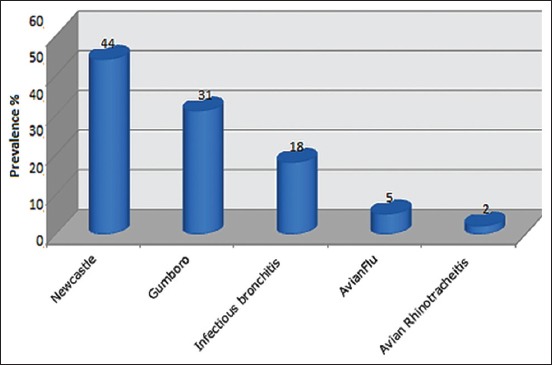
Most common viral diseases in poultry farms.

Data analysis highlights three major viral diseases: Newcastle, Gumboro, and infectious bronchitis disease (Figure-4).

The classification of viral diseases registered has been the subject of veterinary alerts, sufficiently explains the level of infection affecting the poultry farms such as Newcastle, Gumboro, and infectious bronchitis with a rate of 44, 31, and 18%, respectively.

The study also revealed that the occurrence of zoonotic Avian Influenza (5%) and the emerging Infectious Rhinotracheitis (2%) was low in the region. According to the statement of the veterinary inspector of the Wilaya of Batna, authorities recorded a single outbreak of Newcastle in 2015 with over 2160 subjects. Thus, in 2006, a study in Constantine revealed the presence of infectious bronchitis, Gumboro, and Newcastle diseases using at the rate of 83.3, 77.8, and 66.7%, respectively [13].

The earlier findings revealed high rates of occurrence as compared to present findings. This can be explained by the difference in the use of various diagnostic methods. According to the recent surveys [14,15], a total of 418,700 Newcastle disease cases were reported; of which about 275,985 birds died, 73,845 were destroyed, and 580 birds were slaughtered during the 2014 epidemic occurrence of Newcastle disease in Algeria. This confirms in large part the territorial presence of these pathogens.

According to Traore [16], among the viral diseases in Senegal, the main pathologies of traditional poultry farms are as follows: Newcastle disease (the one that causes many problems), variole disease, Gumboro disease, and bronchitis infectious.

### Criteria for selection of drug

**Figure-5:**
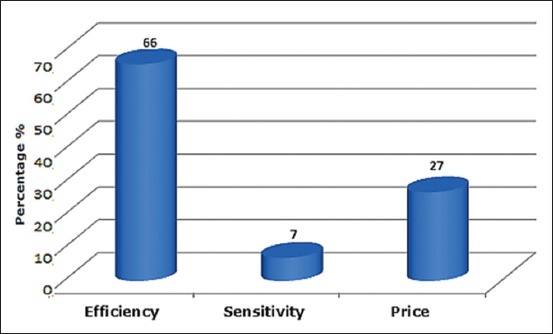
Criteria for medicament selection.

Of the 265 veterinarians interviewed, the choice of the prescribed and/or administered drug, to infected birds, is based on efficacy in 175 veterinarians and on the price in 72 for the other veterinarians. On the other hand, sensitivity represents a small proportion (18 veterinarians) (Figure-5).

On the rational use of antibiotics, it is recommended that the veterinarian should use the results of laboratory analysis on antibiotic sensitivity tests. This makes it possible to target the pathogens, minimizes the cost of the treatment, to the breeders and most importantly, to put in place preventive measures, and allowing less use of antibiotics. However, it is not necessary to perform antibiotic sensitivity because most of the conditions veterinarians can effectively treat many infections probabilistically [17].

### Self-medication in the poultry industry

The results showed that more than 70% of the vets questioned declare that self-medication in the poultry farms is quite often. Our results are very close to those found by Sinaly [18] who found that 79% of breeders follow the practice of self-medication. Hence, they are superior to those of KhalenWouembe [19] who found that 33.64% of breeders in the western region of Cameroon practice self-medication.

## Conclusion

The interpretation of feedback allowed us to conclude that the clinical signs exhibited by the birds and the postmortem changes were commonly used for disease diagnosis by the field veterinarians. The practitioners should be trained and equipped with molecular diagnostic techniques. The bacterial and viral diseases are dominant affections and veterinarians should be made aware of the importance of rational use of drugs in poultry farms.

## Authors’ Contributions

AB and IL collected the data. TK and AK prepared and corrected the manuscript. All authors read and approved the final manuscript.
